# Maternal interventions vigilance harmonization in low- and middle-income countries: Stakeholder meeting report; Amsterdam, May 1–2, 2018

**DOI:** 10.1016/j.vaccine.2019.03.060

**Published:** 2019-05-06

**Authors:** Ajoke Sobanjo-ter Meulen, Flor M. Munoz, David C. Kaslow, Keith P. Klugman, Saad B. Omer, Prachi Vora, Andy Stergachis

**Affiliations:** a Bill & Melinda Gates Foundation, 500 5th Ave N, Seattle, WA, USA; b Department of Pediatrics, Section of Infectious Diseases, Baylor College of Medicine and Texas Children's Hospital, Houston, TX, USA; c PATH, 2201 Westlake Ave, Seattle, WA, USA; d Hubert Department of Global Health, Rollins School of Public Health, Emory University, Atlanta, GA, USA; e Global Medicines Program, Schools of Pharmacy and Public Health, BOX 357631, University of Washington, Seattle, WA 98195-7631, USA

**Keywords:** Pregnancy, Maternal immunization, Pharmacovigilance, Newborn, Vaccine, Safety surveillance

## Abstract

Although major reductions in maternal and child mortality were achieved in the Millennium Development Goals era, progress must be accelerated to meet Sustainable Development Goals health targets by 2030. An estimated 2.7 million neonatal deaths and 2.6 million stillbirths still occur annually. Over the past several years there has been renewed global interest in innovative approaches to maternal immunization to potentially decrease mortality and severe morbidity in neonates, and in the pregnant woman and her fetus. Several new vaccines are in clinical development for indications in pregnant women, e.g., vaccines against respiratory syncytial virus, and group B streptococcus. Achieving near-concurrent introduction of new maternal vaccines in high-, middle-, and low-income countries requires that mechanisms are in place for appropriate safety monitoring worldwide.

The Bill & Melinda Gates Foundation convened a global expert meeting in Amsterdam on May 1–2, 2018, to discuss a framework for appropriate pharmacovigilance for vaccines used during pregnancy based on integrated maternal interventions vigilance (MIV) systems and collection of appropriate data to inform timely decision-making by and for pregnant women. Planning for MIV requires a multi-disciplinary, collaborative approach that fully leverages and builds upon existing resources, and builds new capabilities and capacity where needed. Meeting participants identified priority actions including (1) establishing background rates to better evaluate emerging safety signals and vaccine effectiveness, (2) identifying potential sentinel vaccine surveillance sites, (3) developing data sharing capabilities, (4) creating guidance documents and protocols, and (5) the advanced preparation of culturally-appropriate communication plans and risk management plans.

Integrating MIV across the routine obstetric and neonatal health care delivery continuum and all relevant programs and data systems could result in fundamental improvements in maternal, neonatal and child health. Improved pregnancy pharmacovigilance platforms may strengthen other vaccine and drug product safety systems and improve maternal and child research capabilities in LMICs.

## 1 Introduction

Although major reductions in maternal and child mortality were achieved in the Millennium Development Goals era, accelerated progress is needed especially in sub-Saharan African and Southern Asian countries to meet Sustainable Development Goals health targets by 2030 [1], [2]. An estimated 2.7 million neonatal deaths [3] and 2.6 million stillbirths [4] still occur annually. Lower respiratory infections and sepsis are among the leading causes of neonatal deaths from infectious diseases [5]. The relatively high burden of morbidity and mortality among infants too young to mount an effective immune response or affected by pathogens for which vaccines are not currently available, could potentially be addressed through vaccines administered during pregnancy.

Promising new vaccines for use in pregnancy are progressing to clinical trials. The desired near-concurrent launch of new maternal vaccines in high-, middle-, and low-income countries will require harmonization of maternal interventions vigilance (MIV) efforts, with a particular focus on low-resource settings. MIV refers to safety monitoring of health care interventions, including vaccines and other medicines, received by women during pregnancy. Appropriate pharmacovigilance requires integrated surveillance systems and the collection of appropriate, good-quality data to inform timely decision-making by and for pregnant women.

To this end, the Bill & Melinda Gates Foundation organized a convening of stakeholders (see Appendix I) to discuss MIV harmonization in low- and middle-income countries (LMICs) in Amsterdam, Netherlands, on May 1–2, 2018. The meeting participants consisted of researchers focusing on maternal safety and pharmacovigilance, international regulators and national health authorities, manufacturers developing vaccines for use during pregnancy, experts in global policy and financing, and clinicians and key individuals with professional societies having a specific focus on maternal health. The goal of the meeting was to identify the necessary building blocks for a common MIV framework in preparation for the introduction of new maternal vaccines in LMICs. Presentations and discussions covered the following main objectives of the meeting (i) to align stakeholders on the post-approval safety-related needs along the MIV continuum, (ii) to evaluate the applicability of existing vigilance models to LMICs, (iii) to determine approaches for data sharing, (iv) to establish considerations for developing *a priori* risk communication plans, and (v) to articulate guiding principles for MIV readiness.

## 2 Background

**Fig. 1:**
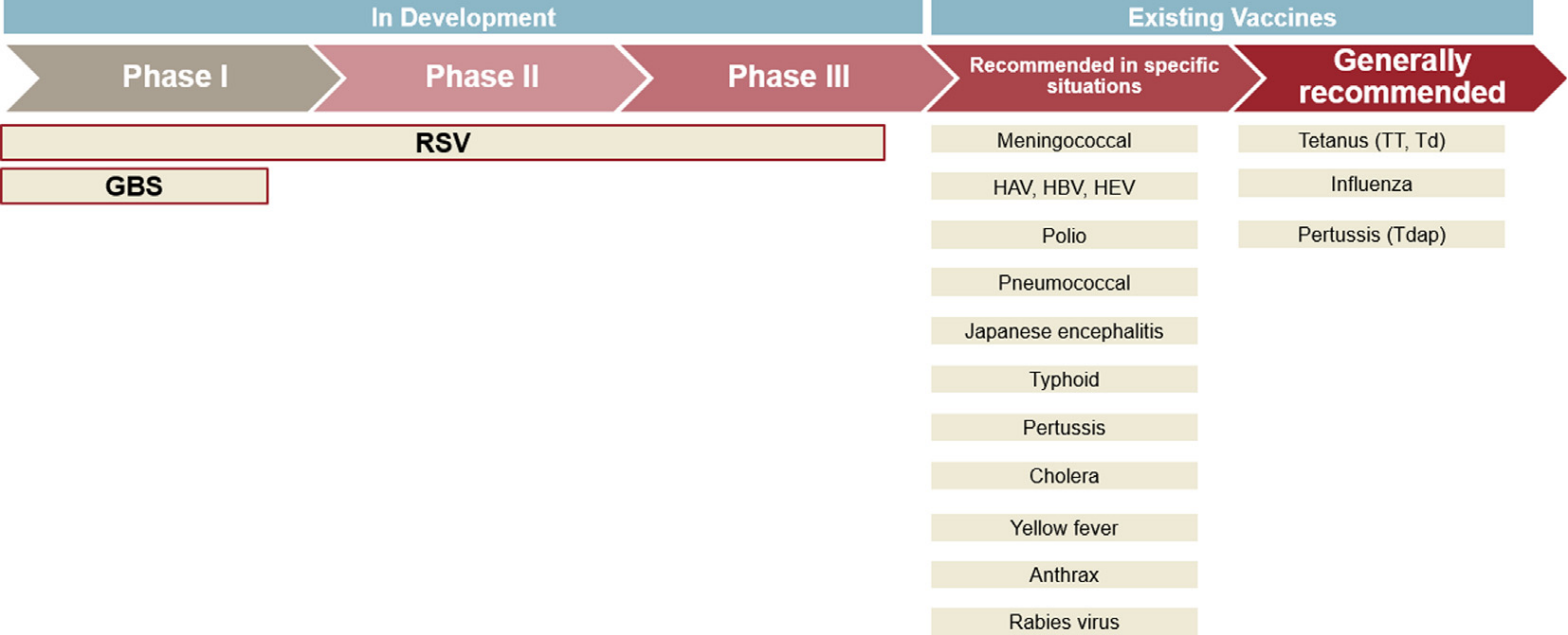
Current and developing pipeline of vaccines for use in pregnant women [11], [12].

While the use of vaccines has led to large reductions in vaccine-preventable disease morbidity and mortality, such programs are limited in protecting newborns and young infants who are too young to receive routine childhood immunizations, and who are affected by certain pathogens for which effective vaccines are not available. Maternal immunization (MI) is the practice of vaccinating pregnant women to provide protection to the mother, fetus, and infant through transplacental transfer of antibodies. MI has the potential to improve maternal and neonatal morbidity and mortality [6], [7]. Fig. 1 shows the current and developing pipeline of vaccines for use in pregnant women. While several vaccines are currently recommended for use in pregnancy, such as inactivated influenza vaccine, tetanus toxoid (TT or with diphtheria toxoid, Td), and acellular pertussis (currently combined with tetanus and diphtheria toxoids, Tdap), clinical development of new vaccines for licensure in pregnant women has only recently made significant progress. Currently, novel maternal vaccines against RSV and GBS have progressed to Phase III clinical trials in pregnant women. Globally, an estimated 319,000 infants are infected with GBS before the age of 3 months with 90,000 resultant deaths [8]. GBS is potentially associated with 57,000 stillbirths [9]. RSV contributes to approximately 34 million annual cases of acute lower respiratory tract infection in children younger than 5 years, with a peak incidence at 10–12 weeks of life [10]. At least one experimental RSV vaccine is on track to be a first-in-class vaccine, and the first vaccine candidate with a primary indication for use in pregnancy to protect young infants from disease. Once the development process is completed, product registration for the RSV vaccine will likely be sought near-concurrently with national regulatory authorities (NRA) in high-, middle-, and low-income countries.

## 3 Aligning on the need for maternal vaccine safety surveillance along the MIV continuum

Traditionally, new vaccines are first launched in high-income countries (HICs) and their launch in LMICs has oftentimes been delayed by several years to a decade or more [13]. Initial introduction into HICs enabled post-approval vaccine safety monitoring through well-established pharmacovigilance systems prior to vaccine introduction in other geographical regions where pharmacovigilance systems are less developed. Potential near-concurrent launch of novel vaccines for the routine immunization of pregnant women in different geographical settings will require that appropriate post-approval safety monitoring mechanisms are in place upon vaccine introduction. Establishing adequate MIV in LMICs is critical to enable appropriate benefit-risk assessment of new maternal vaccines to support their recommendation by health authorities and providers, as well as their uptake and use by pregnant women. Reports of real or rumored adverse events can undermine trust and confidence in vaccines and potentially disrupt routine immunization programs.

Safety information collected during vaccine development is very useful but inherently incomplete and prone to both false negative and false positive signals. Animal testing provides some critical information but is insufficient to predict human safety. Pre-licensure clinical trials are of limited duration and evaluate limited numbers of carefully selected patients in carefully selected settings. Pregnant women are typically excluded from participation in most clinical trials, and pre-licensure vaccine trials in pregnancy typically include women selected to have no or limited underlying pregnancy risk factors. Pre-licensure trials are not designed to detect and characterize infrequent or rare adverse events or evaluate adverse events with delayed onset. Uncertainties in the benefit-risk evaluation during real-world use remain upon product launch. As a consequence, national and international regulators require extensive and specific post-authorization safety monitoring and risk management plans from the product developers for their review prior to product approval. Aside from the manufacturers’ mandate, local, national, regional, and international health interests are best served by comprehensive risk identification, evaluation, management, and communication plans as well. Ultimately, it is the pregnant women and their babies whose health interests are served by robust MIV systems.

Spontaneous reporting systems are a cornerstone of routine vaccine safety surveillance, and the most common pathway to the recognition of adverse events following immunization (AEFI). These are passive systems wherein anyone who uses vaccines, including health care workers, patients or their caregivers, voluntarily report an adverse event to a NRA, a pharmacovigilance (PV) center, or directly to a vaccine or drug manufacturer. For vaccine and drug manufacturers in most countries, adverse event reporting to appropriate regulatory agencies is a regulatory requirement. Spontaneous reporting systems are relatively easy and inexpensive to operate and serve a vital role in detecting potential new safety signals. An example of a passive surveillance system is the Vaccine Adverse Event Reporting System (VAERS), administered by the US Food and Drug Administration (FDA) and US Centers for Disease Control and Prevention (CDC), which collects and analyzes spontaneous reports from vaccine recipients and users, as well as vaccine manufacturers. Since spontaneous reporting relies upon the willingness and capacity of individuals to report, they have several limitations including potentially inconsistent diagnostic criteria, underreporting, a wide range in data quality, lack of denominator data, absence of an unvaccinated control group, and little or no information on background rates. In light of these limitations, the introduction of novel vaccines for special populations and in novel geographies requires additional safety surveillance efforts, including active surveillance.

Active surveillance seeks to ascertain completely the number of adverse events among a group of exposed individuals via a continuous pre-organized process. The cohort of exposed individuals provides the denominator with which a rate can be calculated. Active surveillance can be helpful for the follow-up of potential safety signals identified by passive surveillance. It is especially helpful following the introduction of newly introduced vaccines. The US Food and Drug Administration (FDA) and the European Medicines Agency (EMA) recommend active surveillance for medical products on the market that are likely to be used during pregnancy or by women of childbearing potential [14], [15]. Data identified in such systems can be used to determine rates and risk factors. Examples of active and prospective surveillance include the use of sentinel sites, phase IV cohort studies, and pregnancy registries.

Pregnancy registries may use active or passive systems depending on the protocol. Many national systems use active surveillance, while manufacturer-sponsored registries are more likely to use passive surveillance. Pregnancy registries using a prospective design, i.e., enrolling women at their first antenatal care visit before the outcome of pregnancy is known and following outcomes of women and their children, have the advantages of providing more complete and accurate data and a denominator that can be used to calculate rates. A disadvantage is that they can be expensive to run and to maintain. While pregnancy registries have been used infrequently in LMICs, a successful example of a pregnancy registry was discussed during the meeting. The Assessment of the Safety of Antimalarials during Pregnancy (ASAP) study took place in Mozambique, Kenya, and Burkina Faso. It employed prospective observational methodologies at sentinel sites within health demographic surveillance system programs, allowing for active surveillance and records linkage between drug exposures and pregnancy outcomes, including miscarriages, stillbirths and congenital anomalies [16].

## 4 Evaluating the applicability of existing vigilance models to MIV in LMICs

This session focused on current PV programs in LMICs and their capacities, gaps and opportunities for MIV. While not a systematic review, the three essential steps in the MIV process were discussed, i.e., risk identification, risk evaluation, and risk management and communication [17]. **Risk identification** uses information from spontaneous and active surveillance systems and other sources to identify adverse events in pregnant women and their infants that potentially occur in temporal association with vaccine administration. Locally relevant background prevalence rates of adverse events help put AEFIs in context. **Risk evaluation** involves activities designed to evaluate AEFIs as potential safety signals. Activities such as qualitative evaluation of AEFI cases by expert review committees help to determine if the vaccine could be etiologically related to the adverse event. Active surveillance programs and formal epidemiological investigations such as cohort and case-control studies may be initiated to confirm and quantify the relationship between the vaccine and the AEFI. **Risk management** involves activities associated with the identification, characterization, prevention or mitigation of risks and the measurement of the effectiveness of risk minimization efforts [18]. Risk communication is an exchange of information concerning the existence, nature, form, severity or acceptability of risks. Effective risk communication involves determining the types of information various stakeholders need and want and presenting them in a useful, accessible and meaningful manner. The continuous communication of any material changes in benefit-risk to stakeholders is an essential step. Key stakeholders may include vaccine recipients, health care providers, government officials, vaccine developers, regulatory authorities, and the media. Risk management and risk communication requirements for pharmaceutical companies as stipulated by national regulatory agencies include the preparation of Risk Management Plans, Risk Evaluation and Mitigation Strategies, Periodic Safety Update Reports, Periodic Benefit-Risk Evaluation Reports, and Developmental Safety Update Reports [17].

Each step in the pharmacovigilance process is important. All steps interconnect but require different approaches, tools, and resources. Currently, pregnancy surveillance and pharmacovigilance systems in LMICs are limited by gaps in infrastructure, resources, training, data quality, and methods. In many countries with PV systems in place, low numbers of AEFIs are reported which limits the strength of signal detection, the assessment of safety and the impact of vaccines [17]. Few LMICs allocate budgets to PV resulting in weak systems, a dearth of background rates, and limited use of active surveillance.

## 5 Pregnancy surveillance and pharmacovigilance systems in LMICs

Background rates of pregnancy outcomes and newborn events are essential data in the evaluation of vaccine safety. In the absence of an accurate background rate of an event, it is difficult to know if the AEFI is occurring at an expected rate or at a rate higher than expected. For a novel maternal immunization program, events such as cases of the disease the vaccine is targeting, adverse maternal and fetal pregnancy events (e.g., stillbirth, preterm labor) and newborn events (e.g., jaundice, prematurity) will likely be of special interest.

The public generally holds a heightened concern about maternal and newborn health and safety. An increase in the reported number of serious AEFIs could trigger concerns about the safety of the vaccine. The ability to make a rapid initial assessment of potential safety signals relies upon accurate and relevant background data. Background rates, along with other types of analyses of AEFIs and findings from active surveillance, can provide the public, the media, and public health officials with the data they need to either alert the public health community of a real risk, or to address any inaccurate perceptions about vaccine safety.

In the first meeting session, Current Models: Gaps and Opportunities for Vigilance Efforts in LMICs, existing in-country pregnancy surveillance models were reviewed, including regional examples from India, Kenya, The Gambia, Senegal, Mozambique, Sierra Leone, and Argentina. MIV efforts depend upon the strength of surveillance systems to obtain locally-relevant maternal and newborn morbidity and mortality background rates.

Collaborative efforts by the World Health Organization (WHO), the National Institute of Child Health and Human Development (NICHD), and other programs working on behalf of maternal vaccine safety surveillance were also reviewed. The Global Network for Research on Women and Children (under NICHD) provided examples of registries collecting robust background data. Initiated in 2000, the prospective Pregnancy Registry contains data on over 500,000 pregnancies and pregnancy outcomes. The Maternal Newborn Health Registry, started in 2008, enrolls and follows up on pregnant women and their newborns up to 42 days postpartum. More than 650,000 mother and their infants have been enrolled so far, accumulating data on maternal and neonatal mortality as well as morbidities like miscarriages, stillbirths, and preterm births. Both registries capture data in six countries, including sites in Pakistan, Kenya, Zambia, Democratic Republic of the Congo, Guatemala, and two in India. New and continuing sites are chosen every 5 years.

The Pan American Health Organization (PAHO) uses a network of Latin American Centers for Perinatology in 16 countries in the region. Utilizing a computerized perinatal clinical record with standardized definitions and methods, PAHO built a database that includes maternal demographics, comorbidities, vaccine exposure data, and neonatal outcomes of interest.

The Countrywide Mortality Surveillance for Action (COMSA) program is piloting a partnership with national data collection agencies like the ministries of health, civil registration authorities (birth, death records), and national public health institutes in Mozambique and Sierra Leone. Their ongoing collection of information on births, deaths, and causes of death can play a complimentary role to maternal/newborn-focused data-capture efforts.

Capitalizing on existing systems and sharing good pharmacovigilance practices among stakeholders will help scale-up MIV capacity prior to the launch of the maternal vaccines currently in the pipeline. In addition to identifying potential AEFIs and calculating background rates, they also provide data for use in health programs, policy decision-making, and resource allocation.

Based on these presenters’ experiences and the expertise, gaps and opportunities were identified in small group break-out sessions and then shared and debated in full session. Three primary gaps/opportunities were identified: (i) **the gap in knowledge regarding the existence and capacity of surveillance platforms by country and region**. In response, stakeholders strongly recommended a broad landscape analysis to identify individual county, regional, and national platforms for pregnancy and disease surveillance as well as for pharmacovigilance activities in order to identify potential sentinel sites. Key factors for the review include quality of data, capacity for growth, funding levels, and economic stability; and (ii) **the paucity of coordinated data sharing efforts and abilities**. The standardization of data terminology, a defined data collection scope, and mother-baby record linkage would assist in the harmonization of efforts and systems for the sharing of data. Stakeholders were aware of several organization-sponsored activities addressing data standardization and agreed that including them in, and applying their progress to, the MIV effort adds value and efficiency; (iii) addressing **the absence of background rate data on disease (e.g., RSV, GBS) prevalence and maternal and infant health outcomes** was identified as critical for success. A comprehensive effort will be needed to establish background rates and baseline data to inform MIV safety and efficacy analyses. Leveraging the vaccine developer’s resources for the collection and sharing of epidemiologic data for the calculation of background rates was considered a realistic and valuable opportunity.

## 6 MIV data sharing in LMICs for new maternal vaccines

Pressing needs identified in this session include increased collaboration, access to data, and technology transfer from high- to middle- and low-income countries. Existing platforms could be leveraged to harmonize the collection and aggregation of data but the lack of utilization of standard definitions for maternal outcomes and AEFIs limits current capabilities. Variability in case definitions and diagnostic criteria across data sources (e.g., civil registries, vital statistics), and among differing cultures and languages present challenges for aggregating data. But opportunities exist. The Global Alignment of Immunisation Safety Assessment in Pregnancy (GAIA) Project has been developing standardized definitions, guidance documents, and tools since 2014. Its aims include providing immunization safety researchers with a common language to improve data collection, data analysis, and data pooling, and more effective scientific communication among researchers worldwide, with an emphasis on aiding efforts in LMICs [19]. Adopting GAIA recommendations on terminology, coding, and guidelines and using standardized documents (e.g., protocols, case reports, health records) would facilitate training, capacity-building, and data sharing across regions and countries. Additional systems for case confirmation and case classification are used in diverse settings for the collection of maternal, fetal, newborn and childhood morbidity and mortality. These include the use of International Classification of Diseases (ICD) codes and, for standardized reporting of adverse events, the Medical Dictionary for Regulatory Activities (MedDRA). Harmonization of these case confirmation/classification systems is desirable as well. Collaborations with qualified partners like GAIA, WHO, and others will be necessary, and the work has already begun. Meeting participants recommended using expertise and resources from vaccine developers, universities, and NGOs at each stage of development to design, implement and conduct active surveillance studies.

## 7 Risk communication plans for MIV harmonization

Essential components of all pharmacovigilance efforts include both risk management and risk communication plans to facilitate the collection, evaluation and dissemination of safety information. There are heightened sensitivities associated with maternal immunization due to the vulnerability of both mother and baby. Unidentified safety issues and unevaluated safety signals associated with new vaccines are potential opportunities for misperceptions that can affect vaccine acceptance and uptake. To build trust, the public should be aware of the efforts undertaken to protect their safety. Having a robust risk management plan, communicating about the immunization pharmacovigilance systems, and sharing information gained from the risk management program help to engender public trust.

An example of the importance of being ready to communicate unexpected findings was shared by a presenter from the CDC. An initial evaluation of cases in the Vaccine Safety Database (VSD) at CDC found no increased risk for spontaneous abortions occurring within 28 days of vaccination with the flu vaccine [20]. A review of other studies published before 2015 also found no increased risk [21], [22], [23], [24]. However, analysis of VSD data from the years 2010–2012 found a slightly increased risk, higher than the expected background rate, in women who had received the monovalent H1N1 vaccine during the 2009 influenza pandemic [25]. CDC shared the results with the health care community and the general public so that health care providers could advise their pregnant patients appropriately. A follow-up study to evaluate this potential signal in the VSD system over subsequent influenza seasons is underway with results expected in 2019. The presenter expressed the importance of initially advising key stakeholders of the findings and consulting with them to plan the public communications efforts; then providing timely, comprehensive, and accurate information to address public concerns. The presenter identified the following key aspects of communication messaging when a safety signal is identified:

• Risk communication, when done correctly, can strengthen credibility and maintain trust in immunization programming.
• Partners are an important component of successful communication planning, including the media. Results and findings should be reviewed with key partners before messaging is finalized.
• Communication plans need to include consistent key messages that follow a defined risk communication approach, including: citing facts and data whenever possible, talking about unknowns, providing concrete next steps, and acknowledging both risks and benefits.

Advanced risk management and communication planning are therefore essential to build support for maternal immunization, ensure preparedness for launch, and successfully manage adverse event communications as the need arises. Stakeholder involvement, local context, and harmonization are key elements of both risk and communication planning. Risk management and communication plans should be under preparation well in advance of anticipated product launch (see Table 1).

**Table 1:** Key elements of a maternal immunization risk communication plan in LMICs.

Core content	• Background and context: disease burden, risk and impact of disease, need for prevention for mother and infant
	• Vaccine benefits and identified risks
	• How immunization fits into routine prenatal care
	• Known AEFIs and how to manage them
	• Description of safety system in place for early detection and monitoring of AEFIs
	• Routine and crisis communication plans to address outcomes, rumors, other issues
	• Proactive identification of key roles and assigned responsibilities avoid confusion and multiple uncoordinated messaging; strict adherence to agreed-upon roles

Target audiences	• Key influencers (community leaders, local regulators, Minister/Dept. of Health)
	• Healthcare providers
	• Vaccine recipients and extended family members
	• Traditional media, social media
	• Manufacturers, regulatory agencies, government agencies depending on content

Proposed approach	• Country and context-specific messaging
	• Proactive communications initially with key influencers and decision-makers before public announcements to generate local buy-in
	• Consider brand ambassador to promote the program through media and advertising platforms
	• Crisis communication should come from trusted government official
	• Include all forms of media with coordinated messaging in plain (and multiple) language
	• Include local face-to-face communications
	• Include monitoring and evaluation plan with community updates across all platforms

## 8 Guiding principles for MIV readiness in LMICs

Based upon the key learnings in the prior sessions, the final session challenged the participants to design a Priority Actions List to better achieve MIV readiness for new maternal vaccines in LMICs. MIV should build upon existing efforts, including filling in the framework that has been articulated in previous reports such as the Global Alliance to Prevent Prematurity and Stillbirths (GAPPS) Roadmap [17] and meetings such as the WHO stakeholders meeting on maternal interventions vigilance in Geneva, November 2017 [26].

A focus on the benefit and value proposition for increased MIV capacity was identified as important. While traditional pharmacovigilance is heavily focused on safety, it is also important to incorporate effectiveness into surveillance efforts as an additional priority. Case studies of the health benefits that accrue to the mother, such as potential reductions in hospitalizations for complications of pregnancy and mortality, will enhance the effectiveness narrative. Together with the vaccine benefits to newborns, including potential reductions in incidence of cases, illness severity and mortality, these outcomes can demonstrate value over time and build the value proposition beyond early adopters.

Stakeholders should remain forward-looking and action-oriented to maintain momentum on MIV. It is important to share with the MNCH community that MIV investments will also support their work and to make the case that MIV is a necessary tool for decreasing neonatal mortality and morbidity. Only through strong collaboration will this work be taken to the next level.

Vigilance capacity, systems, and infrastructure already in place should be leveraged. This includes systems for the collection, analysis, and delivery of data and accurate documentation of vaccination. Next steps necessitate identifying and evaluating existing resources and systems and making strategic decisions around where to focus and build further capacity. Gaps in existing ability provide opportunities for novel approaches, e.g., IT devices for AEFI reporting and data collection.

In addition to identifying opportunities, challenges, and priorities, three important cross-cutting themes emerged: (i) **Importance of local context**. Given the remit to evaluate MIV needs for the near-concurrent launch of maternal immunizations in high-, middle-, and low-income countries, participants emphasized the need to ensure local relevance and input at every step of the process, from planning through implementation and in on-going communications. Local community engagement not only builds trust and improves vaccine coverage, it also increases AEFI identification and reporting [17], [27]. Significant local stakeholders include the maternal health care community, health care workers at all levels, and community members – especially those in formal and informal leadership roles; (ii) **Trust is essential** to facilitate uptake and acceptance of maternal immunization. Programmatic success will hinge upon stakeholders’ (e.g., policymakers, pregnant women, health care providers) perception of the favorable benefit-risk balance of maternal vaccination. Robust safety and effectiveness data are needed to enable appropriate decision-making. All aspects of an MIV readiness plan should be built with this in mind and with an emphasis on transparency; (iii) **Time is of the essence.** A number of the recommendations (e.g., obtaining background rates, standardization of case definitions, developing protocols, implementing electronic health records, negotiating data sharing agreements) will take several years to implement. Given the current product pipeline for maternal vaccines, there was consensus on the need to advance on next steps quickly to ensure sufficient readiness for the near-concurrent launch at the time of licensure.

## 9 Summary

Participants discussed requirements for high-quality, integrated surveillance systems, the collection and sharing of appropriate data, and communication strategies to inform timely decision-making by and for pregnant women. Participants also identified a number of recommendations for moving MIV forward, with a focus on immediate next steps to build momentum and prepare for near-term success with a maternal vaccine for RSV. Disseminating the Priority Actions List and identifying priority partners and stakeholders are included in the recommendations in Table 2.

**Table 2:** Key Takeaways, Priority Needs and Actions from the Maternal Interventions Vigilance Harmonization in Low- and Middle-Income Countries Stakeholder Meeting.^a^

Key Takeaways:
The need for MIV is widely recognized and accepted across disciplines.^†^
Improvements in MIV will improve overall pharmacovigilance and MCH systems.
Begin as soon as possible to enable safe and timely vaccine launch in LMICs.^†^

Priority Needs:
Background data on disease and MCH events/outcomes
Implementation and utilization of standardized case definitions of key safety and outcome events
Passive safety surveillance systems (local and regional)
Active safety surveillance programs (sentinel sites in strategic locations)
Communication plan (for vaccine uptake, acceptance, and safety messaging)
Sustainable funding (for MIV infrastructure)

Priority Actions:
Perform landscape analyses to identify:
Existing data systems – gaps, strengths, weaknesses
Potential sentinel sites
Datasets, data-linkage opportunities, data sharing systems

Conduct stakeholder mapping to identify and engage:
Key stakeholder agencies^+^ to endorse Priority Actions and advocate for sustainable funding
Anchor organization to connect MI and MCH stakeholders
Local/national/regional government, health, and community leaders in planning stage

Leverage existing resources in industry, research communities, and international agencies to:
Develop and utilize background rates for disease and pregnancy events and outcomes to evaluate vaccine safety and effectiveness
Adopt standardized definitions to assess and allow comparability of MCH events and outcomes through the work of GAIA, WHO^†^
Adopt standardized AEFI reporting terms based on GAIA, MedRA and ICD-11^†^
Develop product-specific safety surveillance guidance documents and protocols
Develop Communication Plan and Risk Management Plans

a May 1–2, 2018 ^†^Acronyms: MI Maternal Immunization, MIV Maternal Interventions Vigilance, MCH Maternal Child Health, LMICs Low- and Middle-Income Countries, GAIA Global Alignment of Immunization Safety Assessment in Pregnancy, WHO World Health Organization, ICD-11 International Classification of Disease, 11th Revision ^+^For example, WHO, GAVI, SAGE, UNICEF, PATH, and other reproductive, maternal, newborn and child health agencies.

## 10 Conclusion

Pharmacovigilance is an essential component of the regulatory registration and product delivery pathway for all vaccines. Pharmacovigilance for maternal immunizations carries special considerations given the anticipated higher background rates of adverse events in pregnant women and infants, and the low risk tolerance for interventions during pregnancy. For these and other reasons explored at the convening, planning for maternal immunization requires a multi-disciplinary, collaborative approach that fully leverages and builds upon existing resources and invests in new capabilities and capacity where needed. In a Priority Actions List, stakeholders identified priority actions that included: the identification of potential sentinel surveillance sites, the establishment and assessment of background rates of key outcomes to evaluate safety signals and vaccine effectiveness, the development of data sharing capabilities, the creation of standardized guidance documents and protocols, and the advanced preparation of communication and risk management plans.

The goal of good MIV practices is improved mother, newborn, and child health. With MIV for the routine obstetric and neonatal health continuum across all relevant programs and data systems, fundamental improvements in health should result. But the benefits of MIV activities will have an impact beyond maternal and infant health, as improved pharmacovigilance platforms will aid other vaccine and drug product safety systems and improve maternal and child research capabilities as well.

## Conflict of interest statement

Dr. Munoz is site PI for Novavax RSV vaccine at Baylor College of Medicine which provides her with research funding. She is an unpaid member of the Committee on Infectious Diseases of the AAP and the Influenza and RSV working groups at ACIP/CDC. All other authors have no conflicts of interest to declare. All authors materially participated in the planning and conduct of the forum and made substantial contributions to the intellectual content, writing and revising of this article. All authors approve the publication of the manuscript.

## Funding

This forum was supported by The Bill and Melinda Gates Foundation.

## Disclaimer

This report is the summary of the collective views from the meeting participants and does not necessarily reflect the views of Bill & Melinda Gates Foundation and should not be construed as an official BMGF position, policy, or decision unless so designated by other documentation. No official endorsement should be made.

## Figures and Tables

**Table:** 

First	Last	Organization
Steve	Anderson	Food and Drug Administration
Narendra	Arora	International Clinical Epidemiology Network, India
Steven	Bailey	Pfizer
Kathryn	Banke	Bill & Melinda Gates Foundation
Julie	Becker	Rabin Martin
Bonnie	Bender	Rabin Martin
Steve	Black	Cincinnati Children's Hospital
Francesca	Boldrini	Rabin Martin
Louis	Bont	Global Health Group Utrecht Univ.
Nahida	Chakhtoura	US NIH
Hanane	Chaoui	Centre Anti Poison et de Pharmacovigilance du Maroc
Keith	Chirgwin	Bill & Melinda Gates Foundation
Doris	Chou	World Health Organization
Ed	Clarke	MRC Gambia
Amanda	Cohn	US CDC
Clare	Cutland	Chris Hani Baragwanath Hospital
Delese Mimi	Darko	Food and Drug Authority, Ghana
Theresa	Diaz	World Health Organization
Alex	Dodoo	University of Ghana
Michelle	Dynes	US CDC
Linda	Eckert	University of Washington GAIA/Brighton Collaboration
Kathy	Edwards	Vanderbilt University
Jessica	Fleming	PATH
Lou	Fries	Novavax
Raj	Ghosh	Bill & Melinda Gates Foundation
Christine	Halleux	World Health Organization
Deborah	Higgins	PATH
Megan	Huchko	Duke University
Mary Carol	Jennings	Johns Hopkins University
Beate	Kampmann	London School of Hygiene and Tropical Medicine
David	Kaslow	PATH
Nicholas	Kassebaum	University of Washington
Steve	Kern	Bill & Melinda Gates Foundation
Hani	Kim	Bill & Melinda Gates Foundation
Keith	Klugman	Bill & Melinda Gates Foundation
Sonali	Kochhar	Global Healthcare Consulting
Anne	Kruger	Bill & Melinda Gates Foundation
Doris	Kwesiga	Makerere University
Philipp	Lambach	World Health Organization
Jerker	Liljestrand	Bill & Melinda Gates Foundation
James	Litch	GAPPS
Ira	Marto	University of Washington
Flor	Munoz	Baylor College of Medicine
Patricia	Munyalo	Bill & Melinda Gates Foundation
Jennifer	Nelson	Kaiser Permanente
Priscilla	Nyambayo	Medicine's Control Authority of Zimbabwe
Saad	Omer	Emory University
Deepak	Polpakara	John Snow, Inc.
Pratima	Raghunathan	US CDC
Lembit	Rägo	Council for International Organizations of Medical Sciences
Francoise	Renaud	World Health Organization
Alba Maria	Ropero Alvarez	Pan American Health Organization
Rebecca	Schreiner	Bill & Melinda Gates Foundation
Daniel	Scott	Pfizer
Esperanca	Sevene	Universidade Eduardo Mondlane
Mrunal	Shetye	Bill & Melinda Gates Foundation
Kristine	Shields	Shields Medical Writing
Eric	Simoes	University of Colorado
Ajoke	Sobanjo-ter Meulen	Bill & Melinda Gates Foundation
James	Stark	Pfizer
Maria	Stepanchak	GAPPS
Andy	Stergachis	University of Washington
Miriam	Sturkenboom	University Medical Center, Utrecht
Lakshmi	Sukumaran	US CDC
Eugene	van Puijenbroek	Netherlands Pharmacovigilance Ctr
Prachi	Vora	Bill & Melinda Gates Foundation
Niteen	Wairagkar	Bill & Melinda Gates Foundation
Daniel	Weibel	Erasmus University Medical Ctr.
Marc-Alain	Widdowson	US CDC
Adam	Williams	Rabin Martin
Patrick	Zuber	World Health Organization
